# An Integrative Computational Approach to Evaluate Genetic Markers for Bipolar Disorder

**DOI:** 10.1038/s41598-017-05846-4

**Published:** 2017-07-27

**Authors:** Yong Xu, Jun Wang, Shuquan Rao, McKenzie Ritter, Lydia C. Manor, Robert Backer, Hongbao Cao, Zaohuo Cheng, Sha Liu, Yansong Liu, Lin Tian, Kunlun Dong, Yin Yao Shugart, Guoqiang Wang, Fuquan Zhang

**Affiliations:** 1 Department of Psychiatry, First Clinical Medical College/First Hospital of Shanxi Medical University, Taiyuan, 030000 China; 2 Wuxi Mental Health Center, Nanjing Medical University, Wuxi, Jiangsu Province 214151 China; 3 School of Life Science and Engineering, Southwest Jiaotong University, Chengdu, 610031 China; 4 Unit on Statistical Genomics, National Institute of Mental Health, National Institutes of Health, Bethesda, 20852 USA; 5 American Informatics Consultant LLC, Rockville, Maryland 20852 USA; 6 Department of Psychological & Brain Sciences, University of Delaware, Newark, DE 19716 USA; 7 Department of Biology Products, Life Science Solutions, Elsevier Inc., Rockville, MD 20852 USA

## Abstract

Studies to date have reported hundreds of genes connected to bipolar disorder (BP). However, many studies identifying candidate genes have lacked replication, and their results have, at times, been inconsistent with one another. This paper, therefore, offers a computational workflow that can curate and evaluate BP-related genetic data. Our method integrated large-scale literature data and gene expression data that were acquired from both postmortem human brain regions (BP case/control: 45/50) and peripheral blood mononuclear cells (BP case/control: 193/593). To assess the pathogenic profiles of candidate genes, we conducted Pathway Enrichment, Sub-Network Enrichment, and Gene-Gene Interaction analyses, with 4 metrics proposed and validated for each gene. Our approach developed a scalable BP genetic database (BP_GD), including BP related genes, drugs, pathways, diseases and supporting references. The 4 metrics successfully identified frequently-studied BP genes (e.g. GRIN2A, DRD1, DRD2, HTR2A, CACNA1C, TH, BDNF, SLC6A3, P2RX7, DRD3, and DRD4) and also highlighted several recently reported BP genes (e.g. GRIK5, GRM1 and CACNA1A). The computational biology approach and the BP database developed in this study could contribute to a better understanding of the current stage of BP genetic research and assist further studies in the field.

## Introduction

Bipolar disorder (BP) is one of the most common mental illnesses, characterized by alternating periods of depression and elevated mood. Being the sixth leading cause of disability in the world, BP affects approximately 1% of the total population worldwide and about 3% of people in the U.S.^1, 2^. This disease reduces the expected life span by 9.2 years, and about 20% patients with BP commit suicide^3^. Although the cause of BP remains unclear, it has been hypothesized that both environmental and genetic factors play important roles in the development of BP, and multiple genes contribute to risk of the disease^1, 4^.

In recent years, an increased number of studies reported hundreds of genes and proteins related to BP, many of which were suggested as potential biomarkers for this disease, such as BDNF, RELN and ANK3^5–7^. Additionally, several other genes have been studied in clinical trials, such as INS^8^. Moreover, other findings have reported genetic and quantitative changes of genes in connection with BP^9, 10^; both increased and decreased gene expressions were observed^9, 11^. Importantly, many genes were reported to influence BP pathogenesis via unknown mechanisms^12^. Alternatively, some studies have suggested functional mechanisms that can result in the development of BP. For instance, Jang *et al*. found that the genetic dysfunction of TRMP2 causes uncontrolled phosphorylation of GSK-3, which may lead to the pathology of BP^13^.

However, the majority of these BP-gene findings were reported once with no further replication, and over two-thirds were supported by no more than three studies. Moreover, most of these findings came from studies with small sample sizes, which are susceptible to noise. Additionally, owing to variations in data collection and processing approaches, results from different studies were not always consistent. Meanwhile, dozens of new, potentially BP-related genes are reported every year, warranting further validation of these BP candidate genes. While biological experiments can be an effective method of validation, they can, nevertheless, be very costly. To address these issues, we propose in this study a computational biology approach for a systematic evaluation of BP candidate genes.

In recent years, Pathway Studio ResNet relation data have been widely used to study modeled relationships between proteins, genes, complexes, cells, tissues and diseases (http://pathwaystudio.gousinfo.com/Mendeley.html). In this study, we integrated large-scale, BP-related ResNet literature data, independent gene expression data and related pathway/network information to study the functional profile of a large gene pool that has been reported being linked to BP. Our purpose in doing so was to provide an easy-to-use, automatically updating, computational evaluation workflow, capable of generating a BP genetic database (BP_GD); this tool can offer a comprehensive, yet broad perspective that is useful for considering the current stages of BP pathogenesis research at the genetic level. Resulting information revealed that the curated BP target genes were functionally linked to each other, forming a large genetic network to play roles within multiple pathways implicated in BP.

## Methods

**Figure 1:**
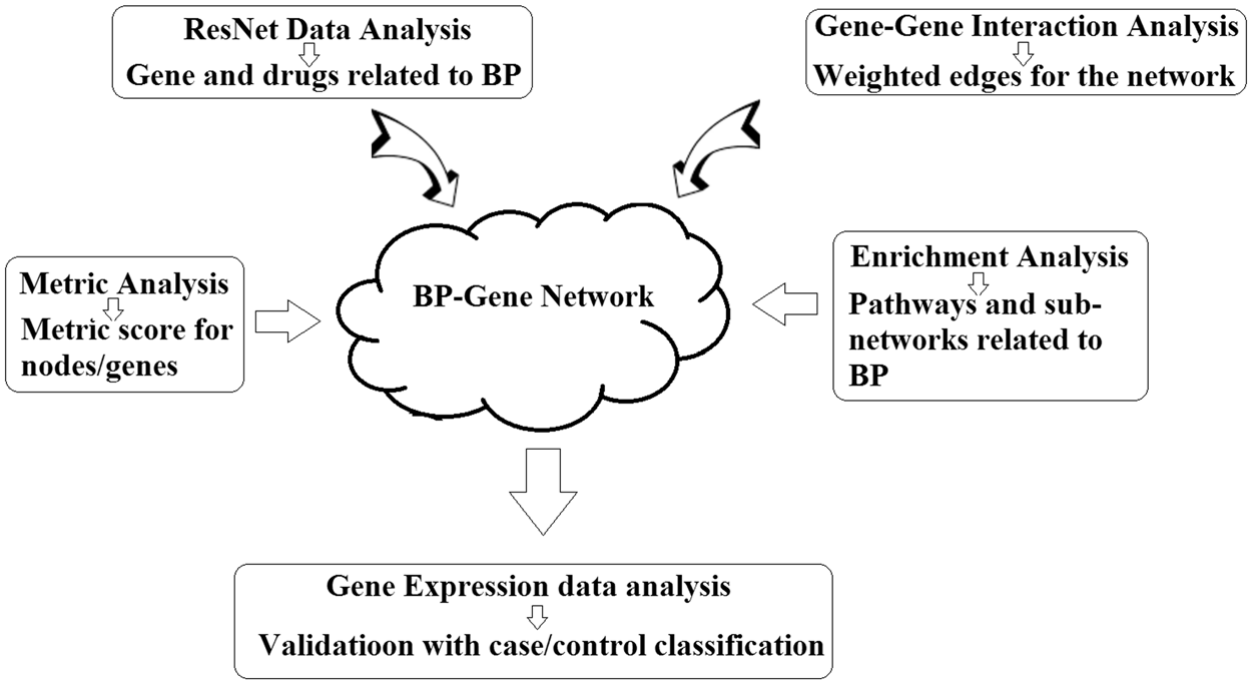
Diagram for the integrated computational marker evaluation approach.

Figure 1 presents the diagram of the proposed computational gene marker evaluation system. The genetic database developed using our approach, BP_GD, has been deposited into an open source “Bioinformatics Database” available at http://database.gousinfo.com, including 535 genes (with metric scores), 198 drugs, 111 pathways and 115 diseases linked to BP. Also included in BP_GD is information on 2,000 + supporting references for BP-gene relationships, 3,000 + for BP-drug relationships and 9,000 for gene-drug relationships, including the titles and relevant sentences where the relations were identified. The BP_GD database is scalable and will be updated monthly or upon request, using our approach.

### ResNet literature data

ResNet relation data (BP-Gene, BP-Drug and Drug-Gene) were acquired from the Pathway Studio ResNet® Mammalian database (http://pathwaystudio.gousinfo.com/ResNetDatabase.html) updated Oct. 2016. The ResNet® Mammalian database is a group of real-time updated literature databases, including curated information on signaling, cellular processes and metabolic pathways, ontologies and annotations, as well as molecular interactions and functional relationships. Modeled relation data are extracted from the 41 M + references covering entire PubMed abstracts and Elsevier and third party full text journals. The ResNet database employs an automated natural language processing-based information extraction system, MedScan, with precision of over 91%^14^. Each relationship data within the database is supported with one or more references. Pathway Studio ResNet Databases is the largest such database among alternatives in the field^15^.

### Enrichment and gene-gene interaction analysis

Pathway enrichment analysis (PEA) and sub-network enrichment analysis (SNEA) (http://pathwaystudio.gousinfo.com/SNEA.pdf) was conducted using Pathway Studio to identify genetic pathways and diseases potentially linked to BP^16^. Building upon this information, a graph theory approach was adopted, to construct a “network” of all relevant genes, which forms the basis for subsequently derived metrics. A pathway-based gene-gene interaction (GGI) analysis was conducted, wherein genes were treated as nodes in a “network” of all genes, and weighted *edges* (i.e. connections between pairs of nodes) reflected relationships within the network. The weight of an edge for nodes A and B is defined as the number of pathways between the two nodes.

### Metrics analysis

Building upon the network framework, we proposed 4 metrics for each gene, including 2 literature-based metric scores (RScore and AScore), and 2 enrichment-based metric scores (PScore and SScore). Justification for these metrics follows from several points. If a gene satisfies the following conditions, it is highly-probable that it shares a meaningful relationship with BP: the gene has been frequently observed in independent studies to be associated with BP (high RScore); the gene plays roles within multiple pathways associated with BP (high PScore); and the gene demonstrates strong functional linkage to many of other genes that were associated with BP (high SSCore). Additionally, we proposed the AScore to present the history of each BP-gene relation and discover novel genes (e.g., AScore = 1 for the genes identified in this year, 2016). Detailed definitions of these metrics are described over the next two sections.

### Literature metrics

The reference score (RScore) of a gene is defined as the number of references supporting a given gene-disease relationship, as shown in Eq. (1).

1 $$R{\rm{Score}}={\rm{The}}\,{\rm{number}}\,{\rm{of}}\,{\rm{references}}\,{\rm{supporting}}\,{\rm{a}}\,relationship.$$

The age score (AScore) of a gene is defined as the earliest publication age of a gene-disease relationship, as shown in Eq. (2).

2 $$A{\rm{Score}}={\max }_{1\le {\rm{i}}\le {\rm{n}}}ArtilcePubAg{e}_{i},$$

where *n* is the total number of references supporting a gene-disease relation, and

3 $$ArtilcePubAge=Current\,date-Publication\,date+1.$$

### Enrichment metrics

Given a disease is associated with a set of genetic pathways ${\mathcal R}$, then the pathway score (PScore) of gene is defined as the number of pathways including the gene (Eq. (4)).

4 $$P{{\rm{Score}}}_{k}={\rm{The}}\,{\rm{number}}\,{\rm{of}}\,\mathrm{pathways}\,\,\mathrm{in}\, {\mathcal R} \,{\rm{including}}\,{\rm{the}}\,k{\rm{th}}\,{\rm{gene}}.$$

The network significance score (SScore) of a gene is defined as the normalized centrality of the gene within the network, as shown in Eq. (5).

5 $$SScore=\frac{N\times {C}_{D}^{W\alpha }(i)}{{\sum }_{j}^{N}{C}_{D}^{W\alpha }(j)}\,,$$

where N is the total number of nodes within the network; ${{\rm{C}}}_{{\rm{D}}}^{W{\rm{\alpha }}}({\rm{i}})$ is the generalized centrality of the i th node^17^, which is defined as Eq. (6).

6 $${C}_{D}^{W\alpha }(i)=de{g}_{i}\times {(\frac{stregt{h}_{i}}{de{g}_{i}})}^{\alpha },$$

where *deg* is the node degree centrality (Eq. (7))^18^.

7 $${C}_{D}(i)=de{g}_{i}=\sum _{j}^{N}{x}_{ij},$$

where N is the total number of nodes, and *i* is the focal node, *j* represents all other nodes; *x* is the adjacency matrix, in which the cell *x*_*ij*_ is 1 if node *i* and *j* are connected, or 0 if not. Note: For network edges built by PGGI, ${C}_{D}\in [0,N]$.

The *strenght* in Eq. (6) is the node strength^19^, defined as the sum of weights of node’s direct ties, i.e.:

8 $${C}_{D}^{W}(i)=stregt{h}_{i}=\sum _{j}^{N}{w}_{ij},$$

where *w* is the weighted adjacency matrix. The cell *w*_*ij*_ is greater than 0 if the node *i* is connected to node *j*, and its value represents the weight of the connection. Note, for network edges built by GGI, ${C}_{D}^{W}\in [0,N\ast M]$, where *M* is the total number of candidate pathways.

In Eq. (6), when 0 < *α* < 1, both high degree and strong ties are favorably measured, whereas, for values of *α* greater than 1, lower degrees and stronger ties are favorably measured^17^, In this study, we set *α* = 0.5, such that the node degree and node strength were equally evaluated.

### Validation using independent gene expression data

We hypothesize that significant BP related genes should contribute to distinguishing BP patients from healthy controls. To validate the effectiveness of the selected genes and the proposed metrics, we performed a Euclidean distance-based multivariate classification^20^ on two independent gene expression data sets (NCBI GEO: GSE35977 and GSE82042)^21^, followed by a leave-one-out (LOO) cross validation, using the overall gene set and the sub-sets selected by different scores as tentative markers. In each run of LOO, gene expression data of all subjects but one are used to train the classifier, which is then applied to the remaining data of the one reserved subject. Permutation was then conducted to test the null hypothesis that a randomly selected gene set of the same size could reach an equal or higher classification accuracy (CR) by chance.

The first mRNA microarray dataset (NCBI GEO: GSE35977) contained gene expression data of 20,044 genes from Postmortem human brain regions of 45 BP patients (24 males and 21 females, aged 44.33 ± 11.41 years) and 50 healthy controls (15 males and 35 females, aged 45.50 ± 8.99 years), with 516 genes overlapping with the curated 535 BP genes (**BP_GD→Related Genes**). The gene expression profile of the second data set (GSE82042) was acquired from peripheral blood mononuclear cells of 193 BP cases and 593 healthy controls; 515 were overlapped with the 535 BP genes.

## Results

### Identification of target genes for evaluation

BP-Gene literature data analysis identified 535 BP candidate genes, supported by 2,047 scientific articles (**BP_GB→Related Genes** and **BP_GB→References for Disease-Gene Relation**). Of these 535 BP candidate genes, 275 (51.40%) have been supported with one reference (RScore = 1), 88 (16.45%) with 2, 59 (11.03%) with 3, 30 (5.61%) with 4, 16 (2.99%) with 5, and 67 (12.52%) with more than 5 references, as shown in Supplementary Fig. S1(a). The gene positions at human chromosomes are presented Supplementary Fig. S1(b), which was generated by using the software R package ‘circlize’^22^. Publication date statistics of the 2,047 supporting references are presented in Supplementary Fig. S1(c), with novel genes reported in each year (Supplementary Fig. S1(d)). Notably, these articles have an average publication age of only 6.4 years, indicating that most were published fairly recently.

### BP-Drug and Drug-Gene relations

BP-Drug and Drug-Gene relation analysis revealed 198 drugs that have been shown evidence of effectiveness in treating BP, supported by 3,115 pre-clinical and clinical study reports (**BP_GB– > Related Drugs** and **BP_GB– > References for Disease-Drug Relation)**. Additionally, 118 out of the 198 drugs have been through clinical trials. These drugs are highlighted in **BP_GB– > References for Disease-Drug Relation** with ‘Object Type’ marked as ‘Clinical Trial’. Supplementary Fig. S2(a) and (b) present the diagrams of the relations between BP and these drugs, with the ones studied in clinical trials shown in (a), and these only studied in pre-clinical in (b). Notably, 444 of the 535 BP candidate genes demonstrate strong drug-gene associations with 193 out of the 198 BP effective drugs, supported by 9,000 references (**BP_GB– > References for Disease-Drug Relation**). Supplementary Fig. S2(c) presents the diagram of the BP Gene-Drug interaction network.

### Enrichment analysis results

PEA showed that, 455 out of 535 genes were significantly enriched within 111 BP candidate pathways/gene sets (p-values < 1e-10, q = 0.001 for FDR; **BP_GB→Related Pathways**). Among these 111 pathways, 17 are related to the neuronal system (with 273 unique genes), 6 to brain function/development (75 unique genes), 5 to behavior (58 unique genes) and 1 to aging (50 unique genes). Due to the lack of space, we only present the top 10 pathways enriched in Table 1 (p-value ≤ 4.5e-36, including 268 out of 535 genes).

**Table 1:** Top 20 Molecular function pathways/groups enriched by 535 reported genes.

Pathway/gene set name	Hit type	GO ID	# of Entities	Overlap	p-value	Jaccard similarity
synaptic transmission	biological_process	0007268	472	112	8.95E-76	0.13
neuronal cell body	cellular_component	0043025	466	94	8.82E-58	0.10
dendrite	cellular_component	0030425	396	87	1.32E-56	0.10
response to drug	biological_process	0017035	509	87	1.19E-45	0.09
synapse	cellular_component	0045202	466	81	2.25E-44	0.09
axon	cellular_component	0030424	318	68	4.69E-43	0.09
memory	biological_process	0007613	76	39	1.51E-40	0.07
postsynaptic membrane	cellular_component	0045211	227	56	7.45E-39	0.08
postsynaptic density	cellular_component	0014069	168	48	1.87E-36	0.07
neuron projection	cellular_component	0043005	378	66	4.54E-36	0.08

Note: For each gene set, the p-value was calculated using Fisher-Exact test against the hypothesis that a randomly selected gene group of the same size (535) would generate the same or higher overlap with a given gene set (q = 0.001 for FDR correction). Jaccard similarity (*J*_*s*_) is a statistic used for comparing the similarity and diversity of sample sets, which is defined by ${J}_{s}(A,B)=\frac{A\cap B}{A\cup B}$, where *A* and *B* are two sample sets.

A SNEA was also performed to identify the pathogenic significance of the reported genes for other disorders that are potentially related to BP. Most of the top 10 diseases identified by the SNEA are also mental health disorders, including schizophrenia, bipolar disorder, major depressive disorder, seizure, Alzheimer’s disease, anxiety, alcoholism, Parkinson’s disease and cognitive impairment. The genes implicated with these diseases present significant overlap with the curated BP_GD genes. The full list of 115 disease related sub-networks enriched with p-value < 1e-50 are presented in **BP_GB→Related Diseases** (q = 0.001 for FDR; 531 out of 535 genes were enriched).

### GGI results

We hypothesized that, if the curated genes are truly linked to BP, they should demonstrate certain functional associations with one another, as they all play roles in BP pathogenesis. To test this hypothesis, we performed a pathway based GGI analysis. Based on the analysis, a gene-gene interaction network was generated. The nodes of the network consist of 455 out of 535 genes that were enriched within the 111 BP target pathways. There were 51,886 edges within the network, the weights of which were defined by the number of pathways shared within each corresponding pair of nodes. The average node strength (sum of the number of genes directly connected) of the network was 211.85, and the node strength for the 80 unconnected genes was defined as 0.

Along with GGI, SScore and PScore were calculated for each gene (**BP_GD→ Related Gene**). The value of a PScore represents how many BP candidate pathways involve a given gene, and an SScore represents how strongly the gene was associated with others in the network.

### Validation results

We hypothesized that, if our selected gene set (535 genes) and, particularly, the highest-ranking genes selected by the proposed metric scores were significant to the pathogenesis of BP, they would lead to significant higher classification accuracy compare to randomly selected genes. To test the hypothesis, classification and LOO cross validation were conducted on two independent public mRNA microarray dataset (NCBI GEO: GSE35977 and GSE82042), followed by a permutation test of 5,000 runs.

**Figure 2:**
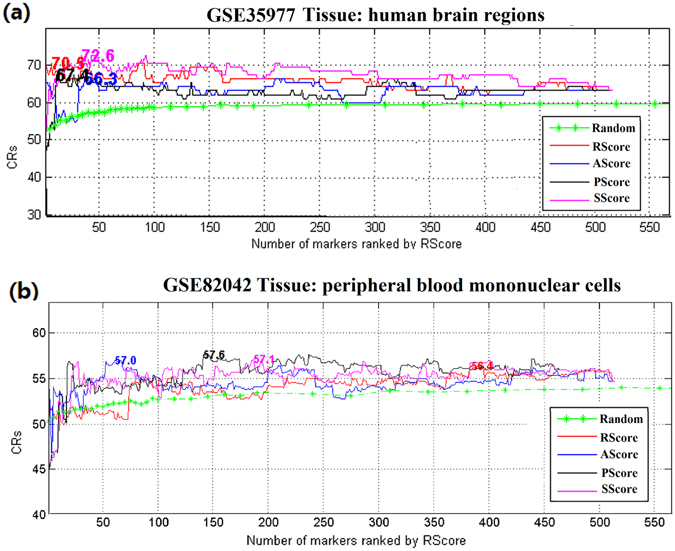
Validation of different metrics through a LOO cross-validation. (**a**) Results from GSE35977. (**b**) Results from GSE82042. Mean CRs of randomly selected genes are displayed in green. The maximum CRs for each metric are presented in corresponding positions.

For the LOO cross validation, we first ranked the 535 genes by different metric scores, then used the highest-ranked *n* (*n* = 1, 2 …) genes as input variables for classification and LOO cross validation. Table 2 and Fig. 2 present the results with the maximum classification ratio (CR) marked for each gene.

**Table 2:** Permutation test on top genes corresponding to highest CRs.

Data Sets	Items	RScore	AScore	PScore	SScore	All Genes
GSE35977 (516/535)	Max CR (%)	70.53	66.32	67.37	72.63	63.16
	#Gene	7	37	11	34	516
	p-value	0.0006	0.046	0.015	0.0008	0.11
GSE82042 (515/535)	Max CR (%)	56.36	57.00	57.63	57.12	54.70
	#Gene	384	61	141	187	515
	p-value	0.073	0.022	0.02	0.034	0.32

Note: p-value in the table refers to permutation p-value, which is defined as the number of runs with equal or higher CRs, using same number of genes divided by the total number of runs.

Figure 2 shows that the highest-ranked genes, selected by their metrics scores (in descending order), were associated with the highest classification accuracy (significantly higher than the average CRs of randomly selected gene sets of the same size). However, adding more, lower-ranking genes did not improve classification.

### Cross-metrics analysis

**Figure 3:**
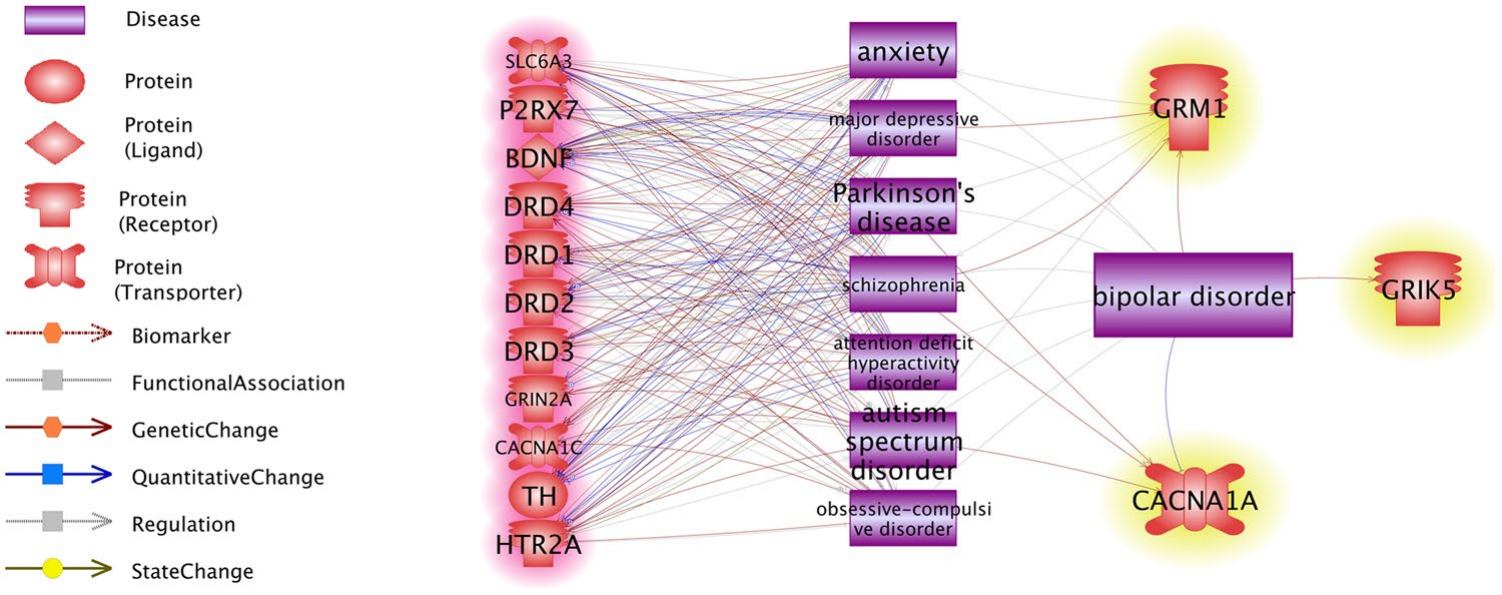
Top BP genes selected by cross-metrics analysis and their relationships to other diseases. The 11 genes that shared overlap in RScore, PScore and SScore groups are highlighted in red; The 3 genes that shared overlap in AScore, PScore and SScore groups are highlighted in yellow. The network was built using the ‘network building’ module of Pathway Studio.

Results from BP classification (Table 2 and Fig. 2) demonstrate that the genes ranked highest by our four metrics led to significantly higher CR compared to randomly selected sets of genes, thus supporting the effectiveness of this approach. To distinguish how literature support and genetic function metrics might operate differently, we selected 55 (out of the total 535) BP-related genes recently published (within the last two years; AScore ≤2) and compared them with an equivalent number of genes ranked highest by each of the other metrics. Cross-metrics analysis (see Venn diagram in Supplementary Fig. S3) indicated that a strong overlap exists between the highest-ranking PScore group and SScore group genes (42/55). Among these 42 genes, 11 were also present in the highest-ranking RScore group, including GRIN2A, DRD1, DRD2, HTR2A, CACNA1C, TH, BDNF, SLC6A3, P2RX7, DRD3, and DRD4. Average scores for each metric were as follows: RScore = 33.55 ± 38.35, PScore = 21.91 ± 7.42 and SScore = 2.05 ± 0.27. Network analysis using Pathway Studio further revealed that these 11 genes also possessed strong correlations with a number of other mental health disorders linked to BP (Fig. 3, highlighted in red). Comparing highest-ranking AScore, PScore and SScore genes, the overlap were: GRIK5, GRM1 and CACNA1A (Fig. 3, highlighted in yellow). Of these, GRIK5 has been only associated with BP, while the other two are also connected to other mental health disorders. (Note, this does not exhaust all possible comparisons; others can also be made using the list of genes and their scores provided in **BP_GD→Related Genes**.)

### Comparison of results to three GWAS meta-analyses

To contextualize the findings of our methods, we compare the BP_GD genes to those found in three large scale BP GWAS meta-analysis. The GWAS data were acquired from top three BP case/control studies curated in Illumina (www.illumina.com), including 9 bio-sets of 30,434 samples in total. Focusing here on several main findings, we provide a more comprehensive overview of all GWAS results in Supplementary Table 1 and Supplementary Fig. S4). Each GWAS identified different genes from one another, with no overlap. However, their identified genes did display overlap with our BP_GD genes (10%, 45% and 31% for meta-analyses 1, 2 and 3, respectively). The lack of overlap between genes identified by these GWAS studies reflects the limitation of using experiment data, alone, for conducting analysis. The results of each study are susceptible to influence from variation in sample sizes, data sources, etc. One final note: the genes identified in these studies were not all identified by our BP_GD. This is due to the fact that some of those genes may not have been reported in the text of the publications (i.e., results only presented in supplementary data) nor been replicated in any other studies.

## Discussion

Studies to date have proposed hundreds of genes associated with the risk of developing BP, with dozens of new candidates identified each year. However, over half of these studies lack replication, and results among the studies are not always consistent. This poses an increasing need for a systematic approach to evaluate the significance of these genes’ relationships to BP. In this study, we worked with large-scale literature data and gene expression data, applying a networks approach to evaluate 535 BP candidate genes. Four resulting metrics were proposed and validated. Based upon these metrics, we developed an automatically updated scalable genetic database (BP_GD) and maintain it online (Bioinformatics Database; http://database.gousinfo.com).

PEA results revealed that most genes within our constructed network were included in pathways previously implicated with BP, including 15 in neurological systems (317/535 genes), 5 in brain function/development (71/535 genes), 4 in behavior (55/535 genes) and 1 in aging (50/535 genes)^23–26^. GGI analysis built upon this PEA data. Results of GGI revealed that the 455/535 nodes displayed a robust connectivity with one another (Supplementary Fig. 3, average node degree: 216.19 edges), supporting the hypothesis that BP candidate genes are functionally linked to one another and across multiple BP-relevant pathways.

In addition to PEA, a SNEA was conducted, which can provide high levels of confidence when interpreting experimentally-derived genetic data against a background of previously published results (http://pathwaystudio.gousinfo.com/SNEA.pdf). SNEA results confirmed that over 97% (522/535) of the 535 genes identified for BP_GD were also identified by previous studies as target genes for multiple other mental health related disorders (**BP_GD→Related Diseases**)^27–29^.

Further, Drug-Gene relation analysis revealed that 444 out of the 535 targeted genes (83.0%) are related to the majority of BP effective drugs (193/198, 96.5%), supported by over 9,000 references (**BP_GD→ References for Drug-Gene Relation**). Additional study of these BP related Drug-Gene relations could contribute to a better understanding of the biological pathogenic mechanisms of BP.

For a quantitative measure of the significance of the 535 selected BP candidate genes, we proposed 4 metrics: 1) publication frequency (RScore), 2) novelty (AScore), 3) number of associated BP candidate pathways (PScore), and 4) network centrality (SScore). We applied these four metrics to a BP case/control classification study, drawing from two independent gene expression data sets (GSE35977 and GSE82042). Results from LOO cross-validation and permutation testing demonstrate that the metrics are significant in gene ranking (Table 2 and Fig. 2). Using the identified gene set as a whole (comprised of 516 and 515 genes out of 535 total, for GSE18123 and GSE37772 respectively) did not significantly predict BP (permutation p-value > 0.1; see Table 2), suggesting that our network metrics were warranted for further analysis of the candidate BP genes. Notably the number of highest-ranking genes, corresponding to the maximum CRs in each of the four metrics, was different across the two data sets. This likely results from differences in between-study variations—such as sample size (ranging from 95 to 786), tissues difference (brain vs. peripheral blood), as well as clinical parameter dissimilarities (e.g., age, gender)—across the data sets. This difference between the two data sets may also stem from variations between subjects’ individual, BP-related genomes^30^.

Cross-metrics analysis showed that 11 genes overlapped across the highest-ranking RScore, SScore and PScore groups (Fig. 3, highlighted in red). This indicates that these 11 genes were frequently identified by different studies to be linked to BP (RScore = 33.55 ± 38.35 references), play roles with in multiple BP candidate pathways (PScore = 21.91 ± 7.42 pathways), demonstrate strong association (SScore = 2.05 ± 0.27 times than that of average). Therefore, our results highlight various aspects that make these genes particularly highly likely to pose biological significance for BP. Moreover, these genes were also identified to play a role within many other mental disorders that were linked to BP, such as schizophrenia, major depressive disorder, autism spectrum disorder, anxiety, Parkinson’s disease, obsessive-compulsive disorder, attention deficit hyperactivity disorder (Fig. 3). Thus, the proposed metric scores collectively support a unique way to focus on a subpopulation of candidate genes that carry especially high promise for illuminating the pathogenesis of BP.

Additionally, 3 newly-reported genes (AScore ≤2) also demonstrated a high SScore and PScore, including GRIK5, GRM1 and CACNA1A (Fig. 3, highlighted in yellow). Although these genes were not frequently referenced in association to BP (RScore = 1 reference for each gene), and presented little or no relation with other BP related mental disorders, they demonstrated high interaction with other genes within the genetic network (SSCore = 2.00 ± 0.18 above average) and are a part of multiple pathways implicated with BP (PScore = 19.00 ± 3.45 pathways). Therefore, our study suggests these, too, may especially warrant further study. In fact, GRIK5 has thus far been reported to influence the neocortical synaptic plasticity^31^, an impairment of which is, indeed, associated with the pathogenesis of BP^32^. The upregulation of GRIK5 has been shown to enhance the release of γ-aminobutyric acid^33^, an effective drug for treating BP^34^. These findings illustrate how our proposed PScore and SScore methods can identify genes that likely hold meaningful, but currently less defined relationships to BP. Therefore, although this study worked with metrics involving prior literature support, it is important to underscore that our PScore and SScore metrics could be applied to any given genes and therefore may contribute to the discovery of novel BP genes.

The genetic database built through our approach, BP_GD, is scalable and can be automatically updated using the computational workflow detailed in this study. Any novel BP-gene/drug relationships will be added as they accrue. Further network analysis incorporating more experiment data may allow for extraction of additional meaningful features. Hence, going forward we will compile more such data, in order to enhance BP_GD’s capabilities for evaluating existing and novel candidate BP genes.

To our knowledge, this is the first study integrating large-scale literature data, experiment data and related pathway/network data for a systematical evaluation of BP target genes. While we validated that such an approach can identify novel information, the study design contained several limitations. First, the target genes were curated from literature reports; a result being reported in literature should not be equated with it necessarily being a true marker for BP. Second, the BP_GD was curated using unsupervised data mining, which is necessary for dealing with such a large volume of literature data (40 + M articles). This method contributes some noise to the data, leading to about a 10% false positive rate for the current database BP_GD. However, given the large scale of our analysis, it is still capable of generating meaningful information, despite this drawback. Therefore, the BP_GD developed in this study is for guidance, to be used as reference in conjunction with expert human judgement. While useful information is certainly generated, users of the database should therefore still make sure to examine the details of derived results.

In conclusion, the computational biology approach adopted in this study offers a novel genetics database tool, based on network algorithms, which can point out new relationships between candidate BP genes and prioritize areas for further inquiry. Due to its comprehensive nature, this approach can lend a wider perspective on emergent themes, which we hope will assist the productivity in the field of BP pathogenesis.

## Electronic supplementary material

Supplementary Information
