# Supplementary material for: An Integrative Computational Approach to Evaluate Genetic Markers for Bipolar Disorder
Source: Sci Rep. 2017 Jul 27;7:6745. doi: 10.1038/s41598-017-05846-4 (PMC5532256; doi:10.1038/s41598-017-05846-4)
Supplement: Supplementary file 1 — Supplementary Information [file 41598_2017_5846_MOESM1_ESM.pdf]

An Integrative Computational Approach to Evaluate Genetic Markers for Bipolar Disorder

Yong Xu[1,*], Jun Wang[2,*], Shuquan Rao[3,*], McKenzie Ritter[4], Lydia C Manor[5], Robert Backer[6], Hongbao Cao[4,7], Zaohuo Cheng[2], Sha Liu[1], Yansong Liu[2], Lin Tian[2], Kunlun Dong[2], Yin Yao Shugart[4], Guoqiang Wang[2], Fuquan Zhang[2]

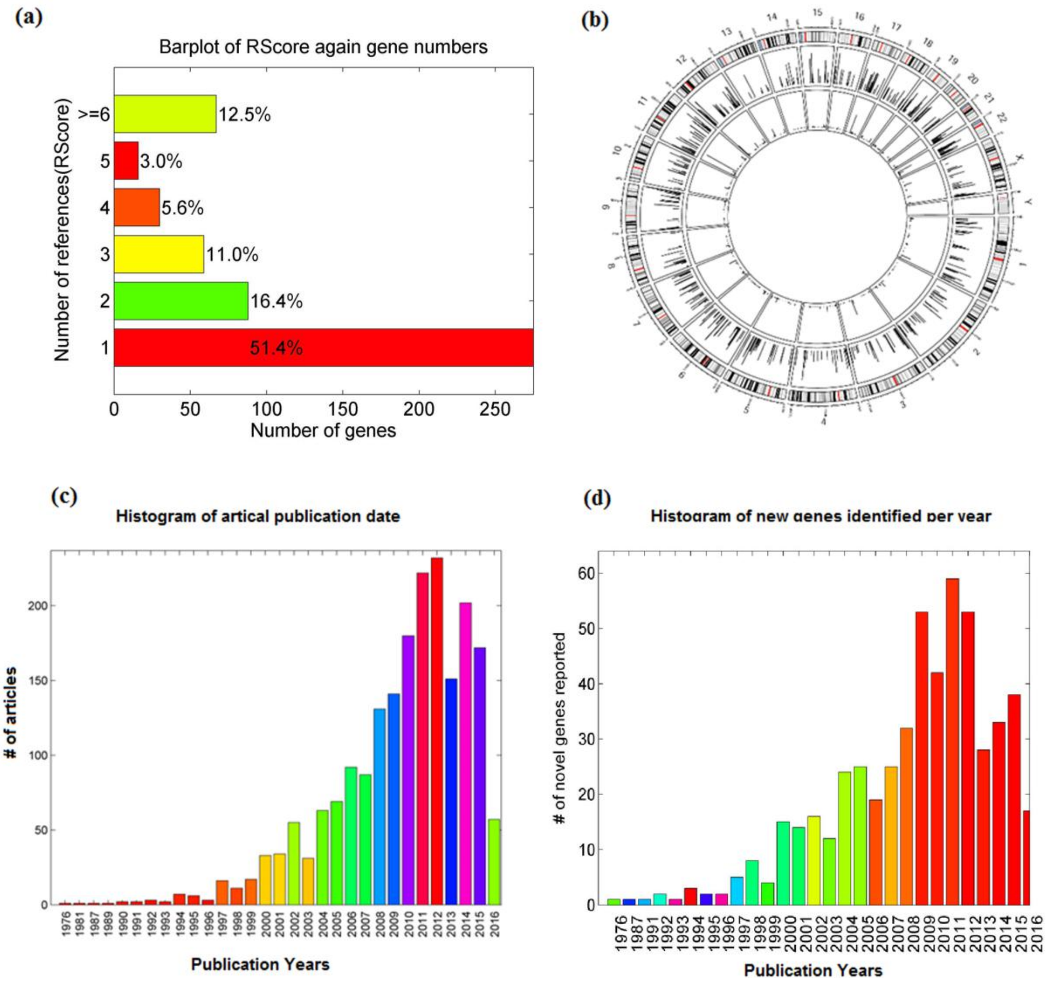

Supplementary Figure S1. Gene-wise relation type distribution of 535 genes and histogram of the publications reporting gene-disease relationships between BP and 535 genes. (a) Number of genes with different supporting references; (b) Ideogram of human chromosome positions of the 535 genes. The inner circle represents the RScores of the genes, and the outer the SSCores; (c) Number of article publications by year; (d) Number of novelty genes identified in each year.

(a)

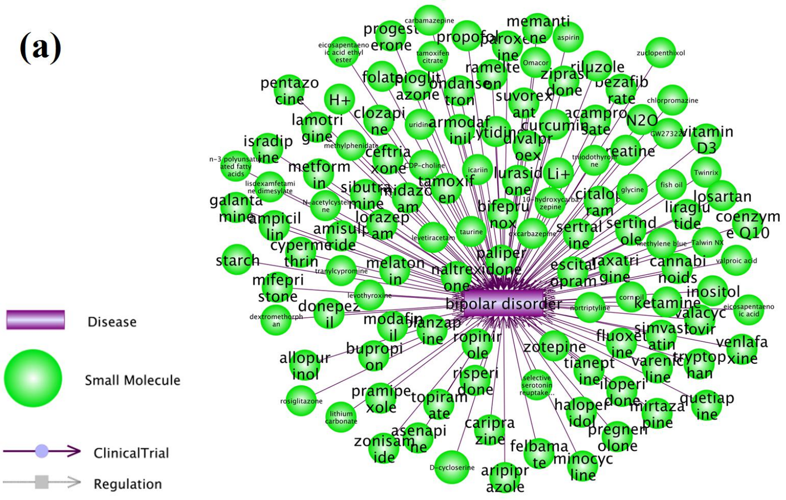

Disease
Small Molecule
ClinicalTrial
Regulation
bipolar disorder

(b)

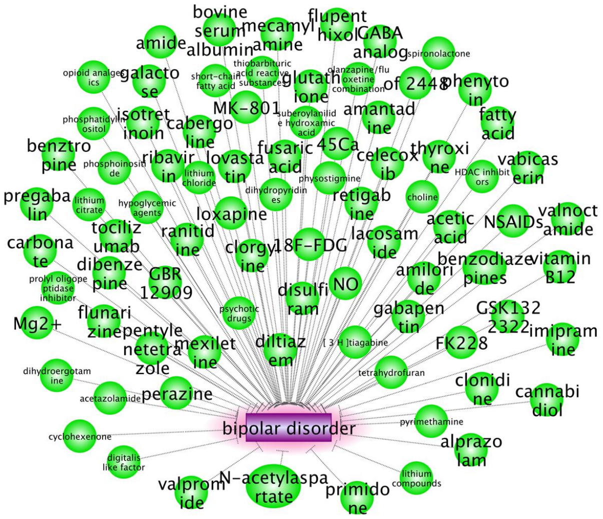

bipolar disorder

(c)

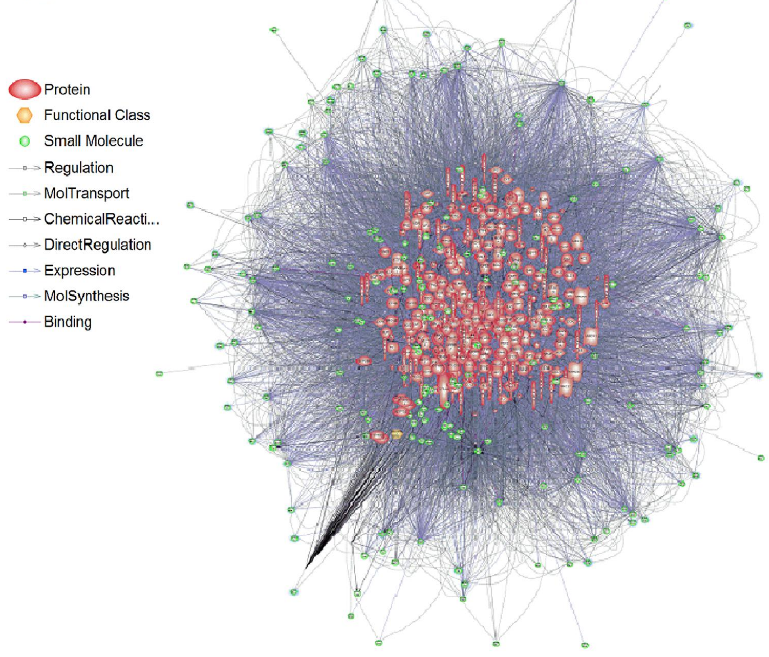

Protein
Functional Class
Small Molecule
Regulation
MolTransport
ChemicalReacti...
DirectRegulation
Expression
MolSynthesis
Binding

Supplementary Figure S2. BP-Drug and Gene-Drug relations. (a) and (b) present the diagrams of the relations between BP and these drugs, with the ones studied in clinical trials shown in (a), and these only studied in pre-clinical in (b). (c) presents the diagram of the BP Gene-Drug interaction network.

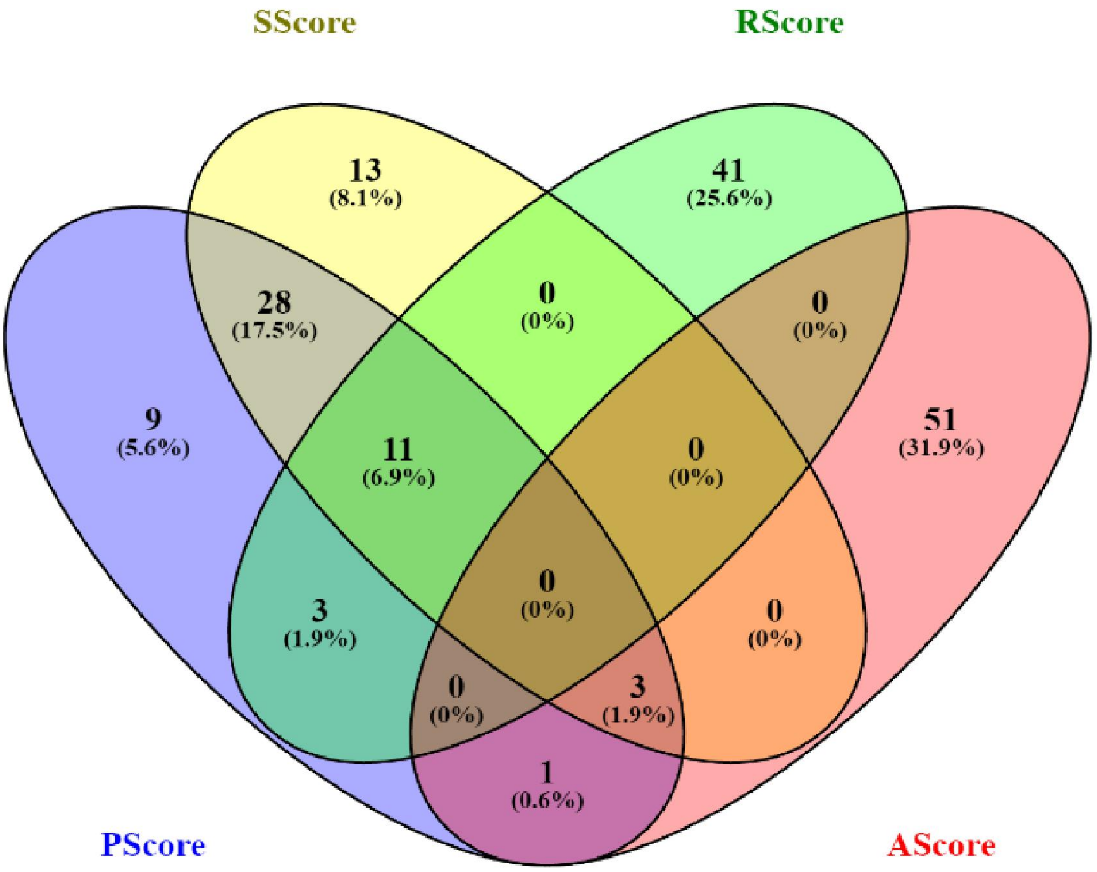

Supplementary Figure S3. Venn diagram of top genes selected by different scores.

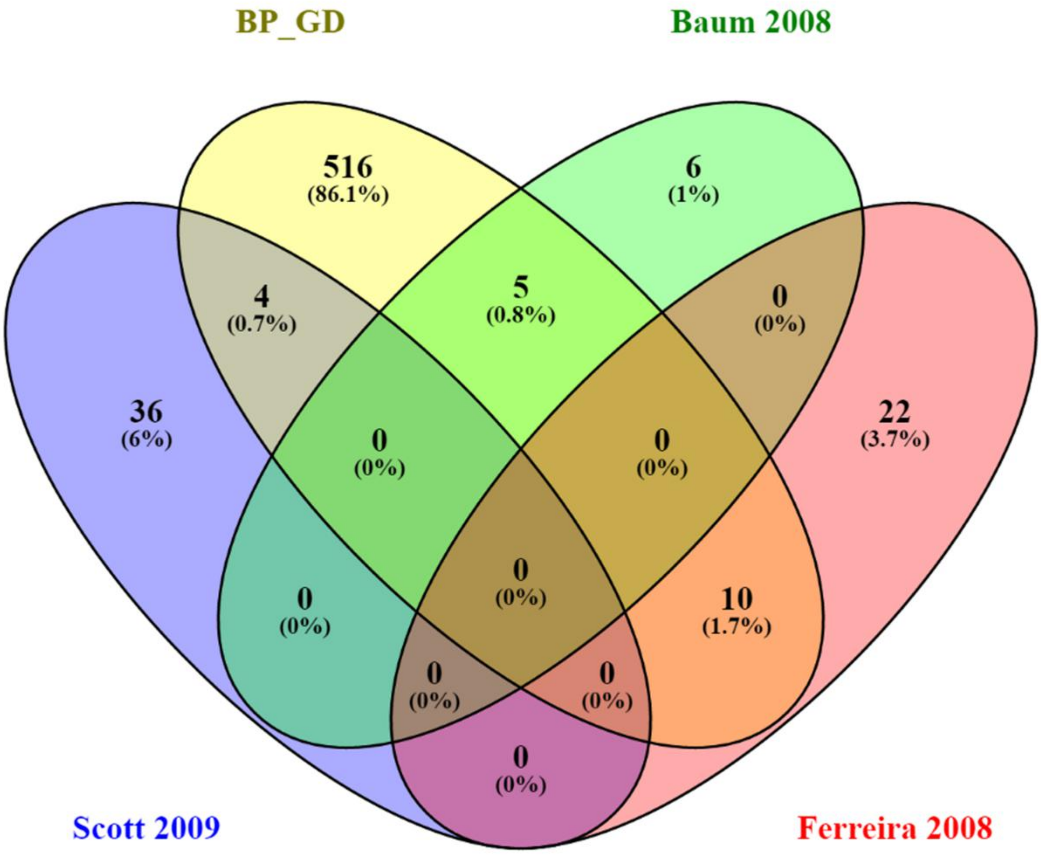

Supplementary Figure S4. Venn diagram presenting the overlap from 3 meta-analyses.

Supplementary Table 1. Comparison to 3 large scale GWAS case/control studies

GWAS Studies	Study 1	Study 2	Study 3
**Data source**	Scott et al. 2009, PNAS	Baum et al. 2008, Mol Psychiatry	Ferreira et al. 2008, Nat Genet.
**Case/control**	3683/14507	772/876	4387/6209
**Sample sources**	Including European ancestry and samples from London, Toronto, and Dundee	Including Caucasians , European ancestry and samples from German	Multiple sources including European ancestry
**# bio-sets**	3	3	3
**Identified Genes**	40	11	32
**Gene Symbol**	ACTN3	ATP6V1G1	ANK3
	ARL4A	DFNB31	ARNT2
	ATP6V1G3	DGKH	C15orf53
	ATP8B3	EDA	CACNA1C
	C1orf87	GALNTL4	CDK1
	CDH11	MAPK6PS3	CMTM8
	CPS1	NALCN	CNNM4
	CTNNA2	NXN	COLEC12
	CTSF	PLSCR4	CRBN
	CYCSP3	RBMS3	DHRS4
	EIF4EP2	SORCS2	DNAH11
	ERBB4		GARNL3
	FSHR		LMAN2L
	GDA		LOC100130207
	GFI1B		LOC100132987
	ITIH1		LOC728288
	KIAA1211L		LOC728667
	KIT		MIR708
	LECT2		NPAS3
	LOC729217		PPIAP7
	LOC730129		PRSS3
	MCTP1		RPSAP19
	MIR181B1		SIAE
	NEK7		SKAP1
	NFIA		SPRED1
	NGFR		SVEP1

	PDGFRA		SYNE1
	PTPRC		TENM4
	PTPRG		THOC1
	REG3A		VENTXP7
	REXO1		ZMIZ1
	RPL10AP3		ZNF385D
	RPL7P13		
	SCIN		
	SI		
	TGFBI		
	TLE3		
	TRDN		
	TSC1		
	UACA		
**Overlap with curated genes in BP_GD**	4 (10%)	5 (45%)	10 (31%)
